# Genomic and Metabolic Disposition of Non-Obese Type 2 Diabetic Rats to Increased Myocardial Fatty Acid Metabolism

**DOI:** 10.1371/journal.pone.0078477

**Published:** 2013-10-21

**Authors:** Sriram Devanathan, Samuel T. Nemanich, Attila Kovacs, Nicole Fettig, Robert J. Gropler, Kooresh I. Shoghi

**Affiliations:** 1 Department of Radiology, Washington University School of Medicine, St. Louis, Missouri, United States of America; 2 Center for Cardiovascular Research, Department of Medicine, Washington University School of Medicine, St. Louis, Missouri, United States of America; 3 Department of Biomedical Engineering, Washington University School of Medicine, St. Louis, Missouri, United States of America; 4 Division of Biology and Biomedical Sciences, Washington University in St. Louis, Saint Louis, Missouri, United States of America; Universidad Pablo de Olavide, Centro Andaluz de Biología del Desarrollo-CSIC, Spain

## Abstract

Lipotoxicity of the heart has been implicated as a leading cause of morbidity in Type 2 Diabetes Mellitus (T2DM). While numerous reports have demonstrated increased myocardial fatty acid (FA) utilization in obese T2DM animal models, this diabetic phenotype has yet to be demonstrated in non-obese animal models of T2DM. Therefore, the present study investigates functional, metabolic, and genomic differences in myocardial FA metabolism in non-obese type 2 diabetic rats. The study utilized Goto-Kakizaki (GK) rats at the age of 24 weeks. Each rat was imaged with small animal positron emission tomography (PET) to estimate myocardial blood flow (MBF) and myocardial FA metabolism. Echocardiograms (ECHOs) were performed to assess cardiac function. Levels of triglycerides (TG) and non-esterified fatty acids (NEFA) were measured in both plasma and cardiac tissues. Finally, expression profiles for 168 genes that have been implicated in diabetes and FA metabolism were measured using quantitative PCR (qPCR) arrays. GK rats exhibited increased NEFA and TG in both plasma and cardiac tissue. Quantitative PET imaging suggests that GK rats have increased FA metabolism. ECHO data indicates that GK rats have a significant increase in left ventricle mass index (LVMI) and decrease in peak early diastolic mitral annular velocity (E’) compared to Wistar rats, suggesting structural remodeling and impaired diastolic function. Of the 84 genes in each the diabetes and FA metabolism arrays, 17 genes in the diabetes array and 41 genes in the FA metabolism array were significantly up-regulated in GK rats. Our data suggest that GK rats’ exhibit increased genomic disposition to FA and TG metabolism independent of obesity.

## Introduction

Type 2 Diabetes Mellitus (T2DM) is a complex, heterogeneous, polygenic disease that affects more than 150 million people worldwide [1]. Recent epidemiological and experimental evidence suggests that there is a close link between the pathogenesis of obesity and T2DM [2], thus confounding the bleak outlook for T2DM. Cardiovascular disease is the leading cause of death among diabetic patients [3,4], attributed to impairment in heart muscle contraction, particularly abnormalities in diastolic function [5]. Although several theories have been put forth, evidence has emerged that severe alterations in myocardial energy metabolism may explain deficiencies in cardiac function among diabetic patients [6]. In particular, insulin resistance in the onset of T2DM shifts the balance of substrate utilization such that the diabetic heart relies almost exclusively on fatty acids (FA) for its energy needs [7]. High rates of FA utilization result in accumulation of myocardial lipids and lipid intermediates leading to lipotoxicity of the heart [8]. Notwithstanding the link between obesity and T2DM, however, it is often neglected that a significant fraction of the population with T2DM, by some accounts 20%, is non-obese [9].

Indeed, obesity is recognized as an independent risk factor for cardiovascular disease [10]. However, numerous studies have shown that non-obese patients with T2DM have a risk of cardiovascular diseases (CVD) similar to that of obese patients [11,12]. In obesity, elevated plasma non-esterified fatty acids (NEFA) levels have been implicated as one of the primary causes for insulin-resistance and downstream consequences [13]. The interplay between increased peripheral NEFA levels, obesity, and diabetes has been studied extensively [14-16]. However, the interplay between diabetes and myocardial substrate utilization in the non-obese state has received limited scrutiny. In a recent work, Desrois and colleagues demonstrated reduced insulin-stimulated glucose uptake in Goto-Kakizaki (GK) rats, a polygenic animal model of non-obese T2DM [17,18]. The authors attributed the decrease in glucose uptake to decreased GLUT4, insulin receptor-β-subunit and insulin receptor substrate-1 protein levels. Given that increased supply and delivery of FA plays a significant role in the pathogenesis of diabetic cardiovascular disease in obese individuals and animal models of obesity, we sought to characterize the interplay between myocardial FA metabolism and cardiac function in a non-obese animal model of T2DM, namely GK rats. To that end, we performed *in vivo* Positron Emission Tomography (PET) imaging to quantify myocardial FA oxidation and utilization in GK rats in comparison to non-diabetic control rats. In parallel, we performed echocardiography (ECHO) with tissue Doppler imaging to assess both cardiac structure and function. We then assayed for the levels of NEFA’s and triglycerides (TG) in both plasma and cardiac tissues. Finally, we performed quantitative PCR (qPCR) arrays on select genes that have been implicated in diabetes and FA metabolism to characterize alterations in gene expression between non-obese GK and non-diabetic rats.

## Materials and Methods

### Animal Model and Study Design

Healthy male adult GK rats were obtained from Charles River Laboratories. The standard Wistar rat serves as the control model for GK rat (Charles River Laboratories, Wilmington, MA). Both GK and Wistar rats were placed under standard housing conditions and fed with commercial pelleted chow *ad libitum*. Unless otherwise stated, all studies were performed at 24 weeks of age. Quantitative PET imaging was performed at two time-points: at 18 weeks of age with Fluorodeoxyglucose (FDG) to characterize myocardial glucose utilization, and at 24 weeks with [^11^C]Palmitate to characterize myocardial FA metabolism. In addition, ECHO imaging was performed to characterize cardiac structure and function. Gene expression analysis was performed to garner mechanistic insight. All experiments were conducted according to a protocol approved by the animal experiment committee at Washington University School of Medicine at Saint Louis (IACUC Animal Welfare Assurance # A-3381-01) and in accordance to ‘Principles of laboratory animal care’ (NIH publication no. 85–23, revised 1985; http://grants1.nih.gov/grants/olaw/references/phspol.htm.

### Rodent PET Imaging Protocol

All animals were fasted overnight (typically 10 hours) before PET imaging. For the imaging experiments, rats (N=4/group) were anesthetized with 1.5 - 2.0 % isoflurane in oxygen. The animals were maintained at physiological body temperature and breathing freely. Dynamic PET acquisition started five seconds following a bolus tail vein injection of the radiopharmaceutical. The imaging protocol consisted of a 20-minute transmission scan to correct for attenuation, followed by a 20-minute dynamic PET acquisition with [^11^C] Acetate (0.6–0.8mCi) to quantify myocardial blood flow (MBF) [14,19] and either 0.6-0.8mCi FDG (at 18wk) or 0.6-0.8mCi FDG [^11^C]Palmitate (at 24wk) as described above. During each imaging session, 5–6 whole-blood arterial samples were collected from the femoral artery to measure whole blood glucose (5 µL), NEFA and TG (20 µL), and insulin (5 µL) levels as well as to correct for the presence of ^11^C metabolites, as described below and in detail by Sharp et al [20]. In total, the PET imaging protocol lasted 2 h. 

### Plasma & Tissue Substrates Determination

Cardiac tissue was isolated, pulverized and NEFA and TG extracted from 1mg of sample using established procedures. All substrates, insulin, and TG levels were measured using commercially available kits by the Nutrition and Obesity Research Center (NORC) or the Diabetes Research Center (DRC) at Washington University School of Medicine (WUSM) per manufacturers’ protocol. 

### Echocardiographic studies

Echocardiograms were performed using non-invasive ultrasound imaging with the Vevo2100 Ultrasound System (Visual Sonics Inc., Toronto, Ontario, Canada) at ages 23-24 weeks as described previously [14]. Briefly, rats (N=4-8/group) were anesthetized with 1% isoflurane and secured to the imaging platform. Complete 2-dimensional, M-mode, and Doppler examinations using a 21 MHz transducer were performed to quantify left ventricular structure as well as diastolic and systolic function. All dimensional measurements were indexed to body weight. Measures of left ventricular structure were also used to determine partial volume corrections performed in conjunction with kinetic modeling of metabolic tracers (described below).

### Pre-Clinical PET Image Quantification

Dynamic images were reconstructed using filtered back projection with a 2.5 zoom on the heart and 40 frames per imaging session. For ease of analysis, all dynamic imaging frames were summed to one image. For each scan, regions of interest were drawn over the left ventricle (LV) blood pool and the anterolateral myocardium to minimize contamination from the liver. Time activity curves were then generated for blood and tissue in each study. To estimate the input function for FDG, [^11^C]Acetate and [^11^C]Palmitate, the hybrid image blood sampling algorithm [21] was employed. 

MBF and myocardial glucose utilization were estimated as described previously [19]. To estimate FA metabolism, a standard four-compartment, five-parameter model (see supplemental material) was used to quantify [^11^C]Palmitate kinetics in the heart [16]. Kinetic parameters K_1_-K_5_ were estimated by optimizing 15 minutes of PET data to the compartmental model. From parameter estimates, intrinsic and extrinsic rates of FA oxidation, esterification and utilization can be calculated (see supplemental material). Intrinsic rates are independent of peripheral effects, i.e., plasma substrate levels, whereas extrinsic rates are associated with flux and absolute accumulation (e.g,, TG) based on the concentration of FA in plasma. Intrinsic measures of FA metabolism are summarized in the Supplemental section. Extrinsic myocardial FA oxidation rate (MFAO), esterification rate (MFAE), total FA utilization rate (MFAU), and tracer extraction fraction (EF), were calculated based on the estimated rate constants (see Supplemental Methods Section).

### RNA Isolation and Real-Time RT-PCR

Cardiac tissues (N=4/group) were harvested and frozen at −80°C until RNA isolation for gene-expression analysis was performed. Total RNA was isolated from heart by using RNEasy Lipid Tissue extraction kit (Qiagen, MD) according the manufacturer’s protocol. RNA concentration, purity, and integrity were determined by spectrophotometric analysis as well as with an Agilent Bio-analyzer. First-strand cDNA was generated by reverse transcription using 1μg total RNA and the RT^2^ First Strand Kit. qPCR was conducted in 384 well plate array format (FA metabolism (PARN-0007E) and Diabetes (PARN-0023E), obtained from SA Biosciences. Real-time RT-PCR was performed using the ABI PRISM 7900 HT system and RT² SYBR Green qPCR Universal master mix with ROX as passive reference dye (SA Biosciences, MD) as per manufacturers protocol. Average readouts of housekeeping genes; *Rpl13a*, *Ldha*, and *Actb* were used for normalizing the data. 

### Statistics and Data analysis

Data processing and statistical analysis was done in either MS Excel or Graphpad Prism (Graphpad software, La Jolla CA). For qPCR analysis, Excel templates provided by SA biosciences were used along with their web analysis tool located at http://pcrdataanalysis.sabiosciences.com/pcr/arrayanalysis.php. A 2-tailed student t-test was performed to test for significant differences between groups. The data is presented as mean ± SD unless noted otherwise. A P-value of p<0.05 was considered significant.

## Results

### Hemodynamics, blood and tissue substrate levels

GK rats exhibited lower weight compared to Wistar rats (Table 1). In agreement with published data [22], GK rats exhibited elevated plasma glucose and decreased insulin levels (Table 1), although the latter was not significantly different, possibly due to the small sample size. GK rats also showed marginally reduced heart rate. While GK rats generally tended to have increased plasma NEFA and TG levels, they were not significantly elevated. However, GK rats exhibited 76% and 57% higher cardiac tissue NEFA and TG levels, respectively. Myocardial blood flow (MBF) estimated using PET imaging, was significantly reduced (P<0.05) in GK rats relative to Wistar rats (Table 1).

**Table 1:** Descriptive data, hemodynamics, and plasma substrate levels.

	**Wistar (n = 4)**	**GK (n = 4 )**
Weight (g)	491.67 ± 9.25	334.17 ± 17.78^a^
Insulin (μu/mL)	14.5 ± 9.98	12.32 ± 3.47
Glucose (mmol/L)	7.80 ± 0.74	9.86 ± 0.94^b^
HR (bpm)	370.33 ± 31.20	323.00 ± 47.69
MBF (mL/g/min)	4.32 ± 0.80	2.46 ± 0.33^b^
Cardiac NEFA (μmol/g of tissue)	0.76 ± 0.08	1.34 ± 0.44 ^b^
Cardiac TG (μg/mg of tissue)	1.74 ± 0.60	2.74 ± 1.23 ^b^
Plasma NEFA (μmol/L)	1084.5 ± 270.10	1434.25 ± 215.88
Plasma TG (μmol/L)	877.01 ± 269.67	1219.97 ± 206.75

Values given as mean ± SD. HR, Heart Rate; MBF, Myocardial Blood Flow.

^a^ P<0.001, ^b^ P<0.05, significantly different than wistars

### Cardiac Structure and Function

Structurally, all anatomical dimensions were indexed to body weight (Table 2). In comparison to Wistar rats, GK rats display increased left ventricle posterior wall thickness during both diastole (LVPWId) and systole (LVPWIs); increased intra-ventricular septal thickness in both the end-diastole (IVSId) and end-systole (IVSIs); higher left ventricular mass index (LVMI, ); and higher left ventricular internal diameter index (LVIDId). Taken together, these small, albeit significant, changes indicated that GK rats developed mild LV hypertrophy accompanied by mild LV chamber dilation. With respect to LV function, fractional shortening (FS), a commonly used parameter of systolic function in rodents, was not significantly different between GK and Wistar groups. In contrast, we observed reduced early left ventricular filling velocity (E) and a reduction in LV filling velocity in the atrial component (A) in GK rats relative to Wistar rats (Table 2). GK rats also had decreased velocity of the early (E’) component of mitral annular diastolic motion. These variations are consistent with diastolic dysfunction. In addition, the Tei index, which is a temporal parameter commonly used to describe global LV function (both systolic and diastolic), was abnormally elevated in GK rats compared with controls.

**Table 2:** Echocardiographic measurements.

	**Wistar (n = 4)**	**GK (n = 4 )**
***Structural***		
LVPWId (mm/kg)	3.69±0.56	4.94±1.00 ^b^
LVPWIs (mm/kg)	5.96±0.72	7.35±1.29^b^
LVIDId (mm/kg)	15.32±2.03	21.91±2.11^a^
LVIDIs (mm/kg)	8.68±1.44	13.60±2.70^a^
LVMI (mg/g)	2.45 ± 0.31	2.84±0.36 ^b^
IVSId (mm/kg)	4.03±0.62	4.80±0.93
IVSIs (mm/kg)	6.58±0.96	7.66±1.26
***Functional***		
FS (%)	43.45 ± 3.56	38.20 ± 8.89
E (mm/s)	1041.33 ± 164.08	818.50 ± 78.10^b^
A (mm/s)	813.67 ± 153.30	560.50 ± 77.88^b^
E/A	1.29 ± 0.11	1.49 ± 0.29
E' (mm/s)	54.43 ± 16.20	36.26 ± 13.54^b^
A' (mm/s)	51.35 ± 12.80	52.27 ± 11.95
E/E'	23.40 ± 10.05	26.84 ± 7.55
IVCT (ms)	10.98 ± 0.98	12.26 ± 1.25^b^
ET	61.51 ± 4.42	65.54 ± 6.23
IVRT (ms)	19.09 ± 3.10	24.88 ± 4.34^a^
Tei Index	0.49 ± 0.05	0.57 ± 0.11^b^

All dimensional measurements were indexed to body weight. IVS, Interventricular septum; IVCT, Iso-volumic contraction time; IVRT, Iso-volumic relaxation time; LVIDId, LV internal dimension index diastole; LVIDIs, LV internal dimension index systole; LVMI, LV mass index; LVPWId, LV posterior wall index diastole; LVPWIs,LV posterior wall thickness index systole. Functional: FS, fractional shortening; E, peak velocity of early diastolic trans-mitral flow; A, peak velocity of late (atria) diastolic trans-mitral flow; S', peak velocity of systolic mitral annular motion; E', peak velocity of early diastolic mitral annular motion; A', peak velocity of late (atrial) diastolic mitral annular motion; IVCT, iso-volumic contraction time; ET, LV ejection time; IVRT, iso-volumic relaxation time; Tei Index, LV performance index calculated as (IVCT+IVRT)/ET. Values given as mean ± SD (N=4-8/group). ^a^ P<0.001, ^b^ P<0.05, significantly different than wistar rats.

### In vivo FA metabolism

**Figure 1:**
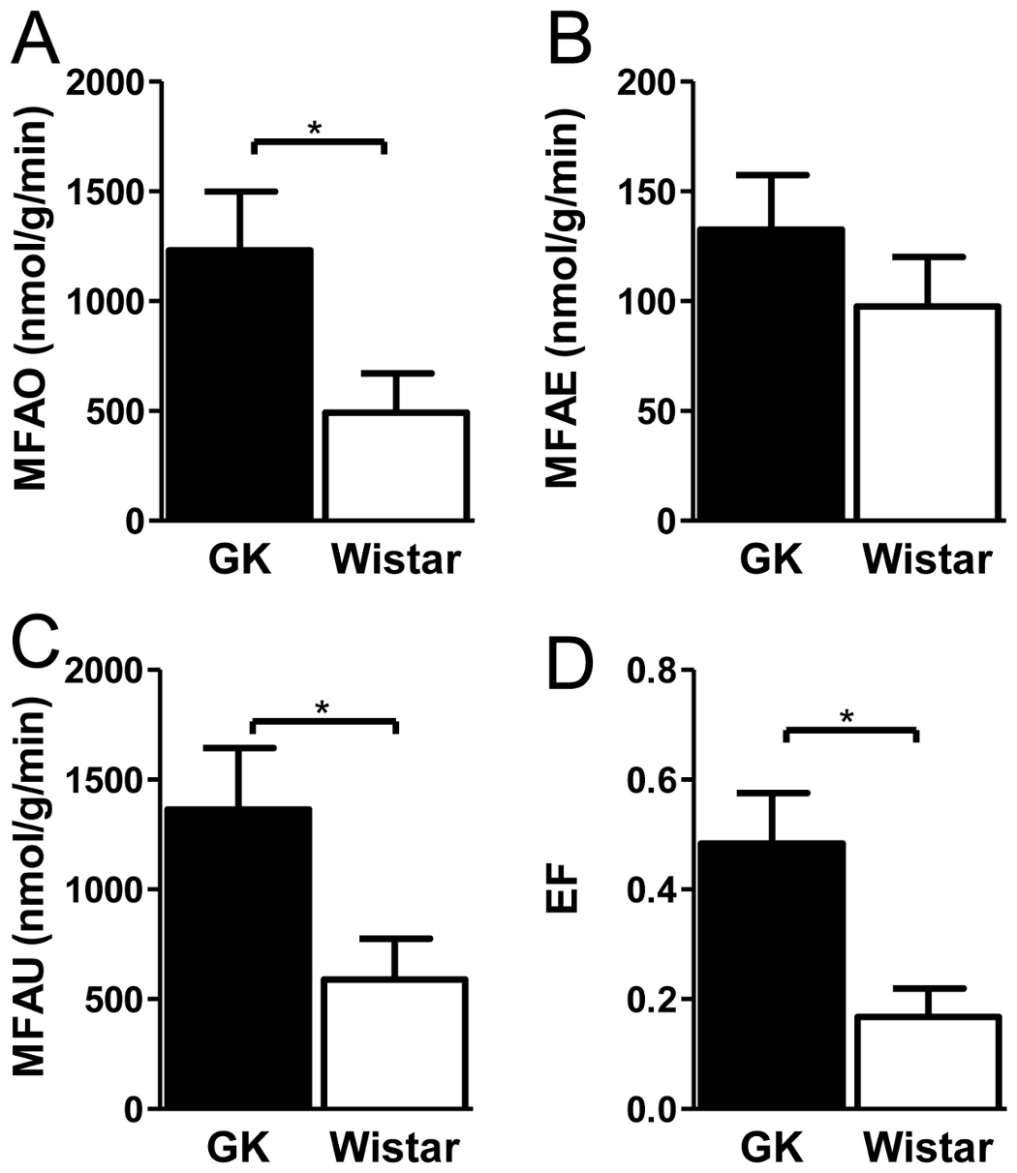
PET measures of myocardial fatty acid metabolism and blood flow. (**A**) Extrinsic myocardial fatty acid oxidation rate (MFAO) (**B**) Extrinsic myocardial fatty acid esterification rate (MFAE), (**C**) Extrinsic myocardial fatty acid utilization rate (MFAU), (**D**) myocardial Extraction Fraction (EF) in GK and control rats. *denotes that GK rats are significantly different (P<0.05) than Wistars for that measurement. All results are presented as mean ± 1 SEM with N=4/group.

GK rats exhibited nearly two-fold higher extrinsic MFAO compared to Wistar rats (Figure 1A) with a marginal increase in MFAE (Figure 1B) indicating that the majority of the increased MFAU (Figure 1C) is attributed to FA oxidation. Intrinsic measures of myocardial FA metabolism are provided in the Supplementary section, but generally display a pattern similar to that of extrinsic measures. Though MBF was almost twice as low in GK rats compared to Wistars, we observed nearly two-fold increase in the EF of FA, i.e. the flux of FA utilized normalized to that of MBF, as measured by PET (Figure 1D). 

### Gene Expression Analysis Panel

Differences in the expression of genes involved in FA metabolism and diabetes were analyzed by qPCR. Statistical analysis of the qPCR data yielded a list of significant genes (P<0.05) for FA metabolism and Diabetes panels. The heat-maps described below depict the fold change and their functional grouping.

#### Expression profile of genes involved in Diabetes

**Figure 2:**
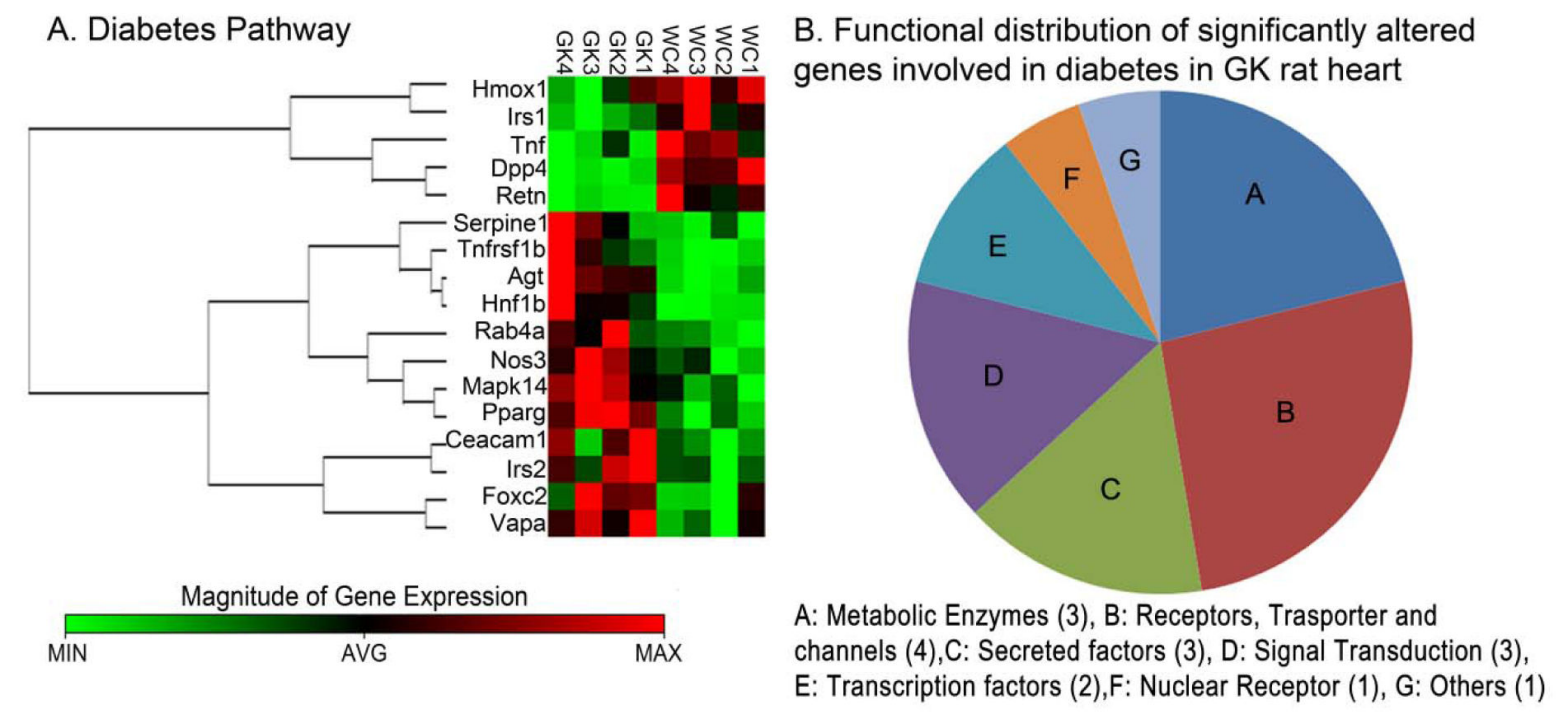
Clustering and functional grouping of genes involved in diabetes. (**A**) Heat map and Gene correlation clusters of genes involved in diabetes including; transcription factors, metabolic enzymes, Receptors and secreted factors. Gene correlation clusters were calculated using the Spearman Correlation Distances and complete linkage for hierarchical clustering. Data for individual biological replicates are shown at the 95% or above confidence level. (**B**) Pie chart illustrating functional distribution of genes involved in diabetes and varies between GK and Wistar rats. (N=4/group).

**Figure 3:**
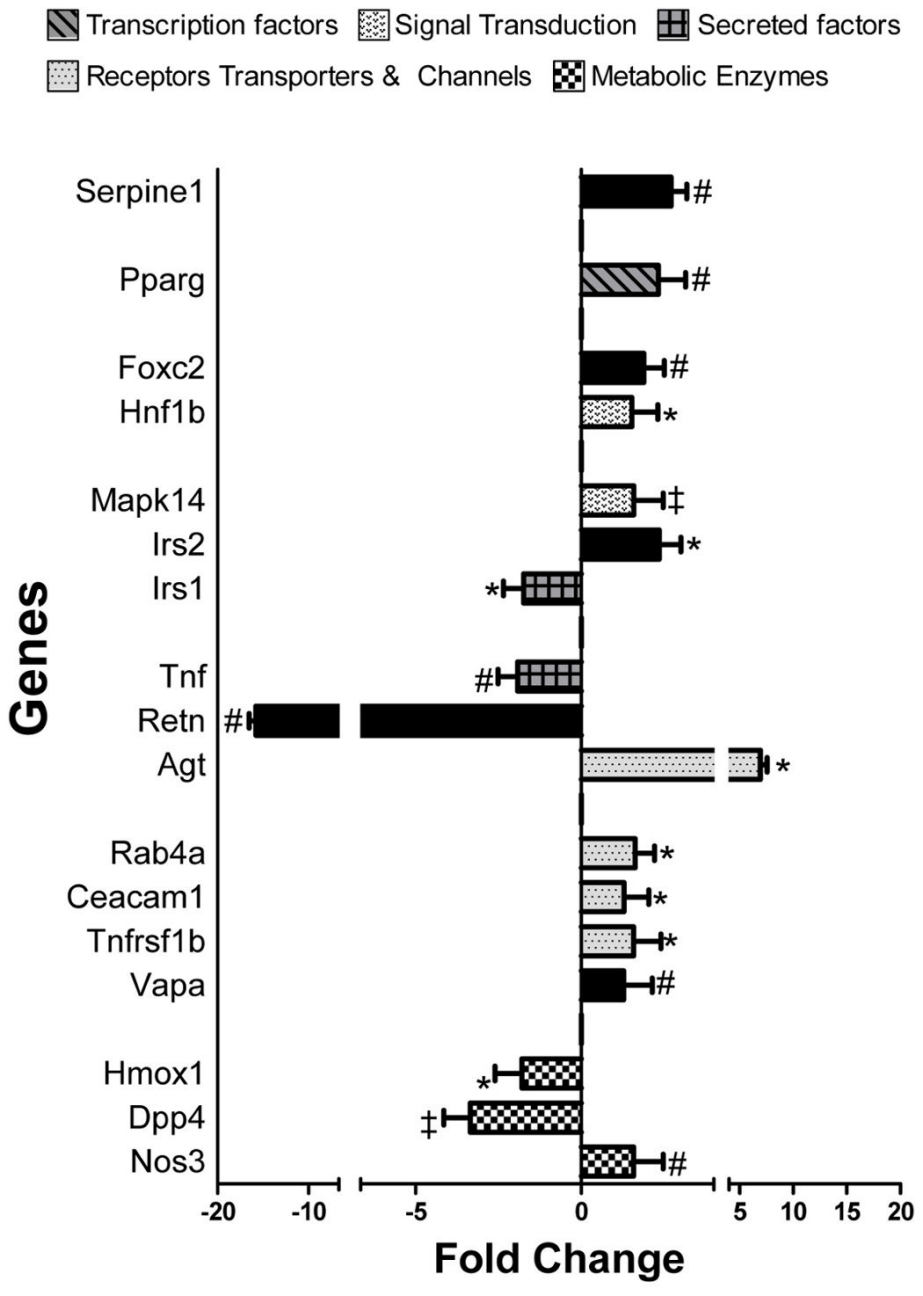
Differential expression of genes involved in diabetes. RNA from N=4/group rats were isolated and expression analyzed using PCR array from SA Biosciences. All results are presented fold change (mean ± 1SD) relative to Wistar rats. *P<0.05, #P<0.01 ‡P<0.001 denotes significance (Students’t-Test).

In the diabetes array (Figure 2), 17 genes were significantly altered between GK and Wistar rats. These significantly altered genes represent diverse functional categories including metabolic enzymes, signal transduction, receptors, and secreted factors. Notably, resisitin (*Retn*) was down-regulated by more than 10-fold in GK rats. We also observed that, Insulin receptor substrate 1 (Irs1), dipeptidyl-peptidase (*Dpp4*) and Heme oxygenase1 (*Hmox*) were down-regulated at varying levels, while genes *Mapk14, Nos3, Agt, Hnf1b Pparg* exhibited up-regulation in comparison to Wistar rats (Figure 3). 

#### Alterations of genes involved in FA metabolism

**Figure 4:**
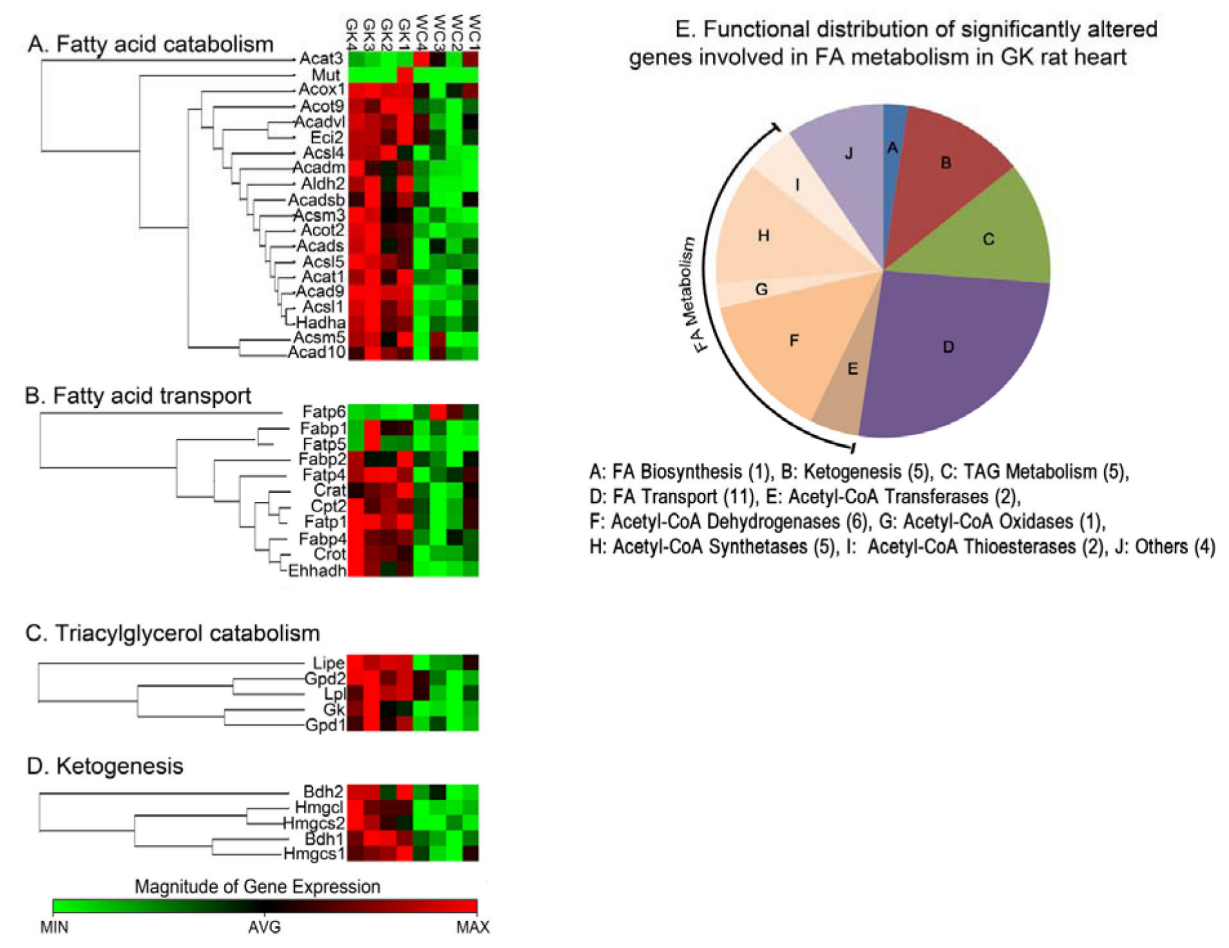
Clustering and functional grouping of genes involved in fatty acid metabolism. (**A**-**D**) Heat map and Gene correlation clusters of genes involved in FA Metabolism including; fatty acid catabolism(**A**), fatty acid transport (**B**), triglyceride biosynthesis (**C**), and ketogenesis (**D**). Gene correlation clusters were calculated using the Spearman Correlation Distances and complete linkage for hierarchical clustering. Data for individual biological replicates are shown at the 95% or above confidence level. (**E**) Pie chart illustrating functional distribution of genes involved in FA metabolism in heart and varies between GK and Wistar rats. (N=4/group). .

**Figure 5:**
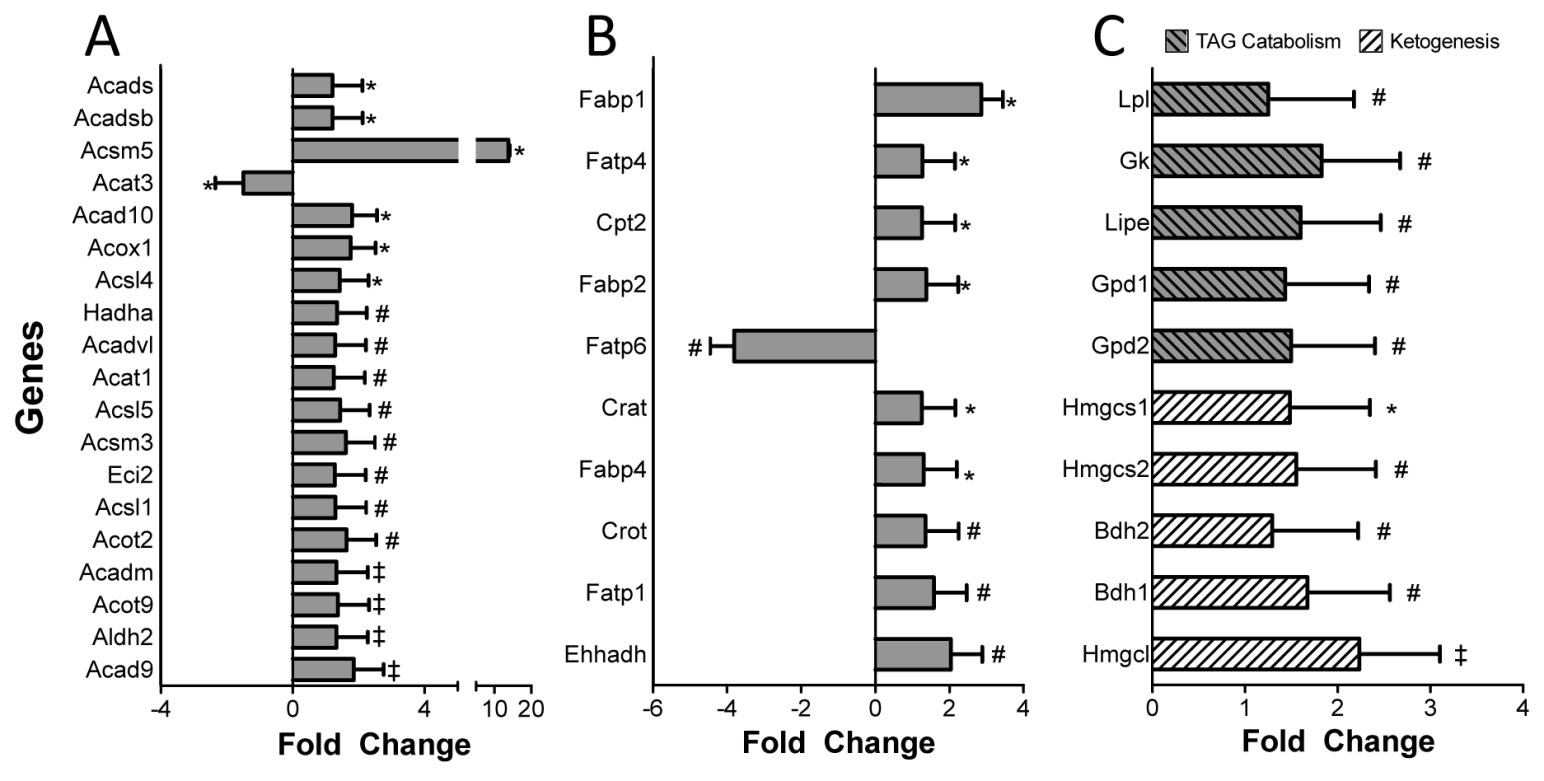
Differential expression of genes involved in fatty acid metabolism. RNA from (N=4/group) rats were isolated and expression analyzed as mentioned earlier. The fold changes of genes grouped based on their function; Fatty acid transport (**A**), Fatty acid catabolism (**B**), Ketogenesis and TG metabolism (**C**), are presented here. All results are presented fold change (mean ± 1SD) relative to Wistar rats. *P<0.05, #P<0.01 ‡P<0.001 denotes significance (Students’t-Test).

We analyzed the expression of genes involved in FA transport, activation, and the metabolic fate of FAs in GK rats. The analysis indicated that 41 of the 84 genes involved in FA metabolism were significantly altered (mostly up regulated) in GK rats in comparison to Wistar rats (Figure 4). In particular, several genes involved in FA transport and activation, (Acyl-CoA synthetases, Fatp’s, Fabp’s, transferases) (Figure 5A); β-oxidation, (dehydrogenases, and thioesterases) (Figure 5B); TG biosynthesis and degradation, and ketogenesis (Figure 5C) display elevated level of expression in GK rats compared to Wistar rats. 

## Discussion

Epidemiological studies suggest a close link between obesity and the increased incidence of T2DM in the USA and worldwide. Accordingly, various animal models of obesity have been reported and used in studying the interplay between obesity and T2DM [23]. To study T2DM independent of obesity, we utilized GK rats—a polygenic animal model of T2DM—with particular emphasis on interplay between T2DM and myocardial FA metabolism and function. We performed ECHO to assess cardiac function, quantitative PET imaging to characterize FA metabolism, and qPCR to provide a mechanistic insight on the functional, metabolic, and genomic myocardial phenotype of GK rats. 

### In Vivo Functional and Metabolic Imaging

Echocardiographic measurements suggest that GK rats develop mild structural remodeling of the LV and impaired cardiac function in agreement with previously published reports on GK rats [18,24]. In previous studies, and confirmed within, GK rats exhibited reduced glucose uptake, which was attributed to deficiencies in insulin signaling [17]. We performed *in vivo* PET imaging to quantify both intrinsic and extrinsic measures of cardiac FA metabolism [14,16]. The data suggest that GK rats have higher FA utilization rates in comparison to Wistar rats. The higher rate of FA utilization is attributed primarily to higher FA oxidation rates. In addition, GK rats exhibit higher MFAE, albeit not significant, in comparison to Wistar rats, while cardiac TG was significantly higher in GK rats in comparison to Wistar. While both cardiac TG measures and PET-derived measures of MFAE exhibit similar trends, the discrepancy in the magnitude can be explained by several factors: 1) in estimating MFAE, it is assumed that the metabolism of FA with various chain-lengths is similar to that of C16 palmitate, which may not necessarily be the case. In fact, oxidative substrate chain-length preference has been documented [25,26]; and 2) cardiac TG content is the sum of multiple sources of FA, including those derived from plasma and those broken-down from plasma TG and re-esterified in tissue. PET measures of MFAE, however, primarily reflect metabolism of plasma FA since [^11^C] Palmitate is introduced in the bloodstream. FA derived from lipolysis of plasma TG may also be reflected in MFAE, depending on functional pools of available NEFA. Taken together, *in vivo* data suggests that GK rats have an innate preference for FAs in comparison to Wistar rats. To gain mechanistic insight for differences in FA metabolism between GK and control rats, we performed qPCR on a panel on genes implicated in diabetes as well as select genes involved in FA metabolism.

### Differential Expression of Genes Implicated in Diabetes

Expression analysis of genes implicated in diabetes indicates differential expression of several genes encoding receptors, transcription factors, and metabolic enzymes. Of particular note is resistin, a hormone that links inflammation, insulin resistance and coronary heart disease [27], which is down-regulated more than 10-fold in GK rats. The heart is thought to be a resistin target where it enhances phosphorylation of insulin receptor substrate-1 and thus insulin resistance, impacting glucose transport [28]. It has been observed that resistin’s action is modulated, at least in part by TNF-α [29], which is also down-regulated in our analysis. We observed down-regulation of *Dpp4* which is known to alter post-prandial lipid levels [30]. *Agt*, coding angiotensinogen involved in production of Ang I and subsequently Ang II, which has been shown to regulate triglyceride biosynthesis, is up-regulated [31]. Taken together these data support the notion that the cardiac tissues in GK rats have an altered metabolic preference. 

### Differential Expression of Genes Involved in FA Metabolism

Activation of FAs is a key step in FA oxidation and is catalyzed by acyl-CoA synthetases. Carnitine palmitoyl transferase 2 (CPT2), Carnitine acetyl transferase (CRAT), and Carnitine O-octanoyl transferase (CROT) are key enzymes involved in activated FA transport to the mitochondria. We observed increased expression of these genes along with several other FA transporters (Fabp’s and Fatp’s). Post entry into the mitochondria, chain-length specific Acyl-CoA dehydrogenases (ACADs) carry out the first step of mitochondrial beta-oxidation. Indeed, we observe increased expression of several ACADs in agreement PET measures of MFAO. Gene encoding for lipoprotein lipase (LPL), which is involved in the hydrolysis of lipoproteins, is similarly up-regulated in the GK rats along with hormone-sensitive lipase (HSL). Conversely, Gpd1, Gpd2 and Gk which are involved in TG biosynthesis are up-regulated as well. Combined increase in genes involved in TG hydrolysis and biosynthesis suggests continuous lipolysis and biosynthesis of TG, as observed previously [32]. Moreover, in addition to plasma NEFA as a source for TG formation, hydrolysis of plasma TG by LPL may further contribute to the cardiac TG pool resulting in an overall net accumulation of TG in the heart. Accordingly, we observed increased MFAE, notwithstanding potential discrepancies between PET measures of TG and cardiac TG discussed above. In conclusion, our data suggest increased mobilization of resources resulting in increasing FA metabolism. Interestingly, we observed increased expression of genes involved in ketogenesis, namely *Bdh1, Bdh2, Hmgcl, Hmgcs1*, and *Hmgcs2*. Ketone bodies such as acetoacetate and β-hydroxybutyrate predominantly act as water soluble equivalents of FA to power peripheral tissues like heart and skeletal muscle. Increase in ketone metabolism may be a feature of the failing heart, as has been demonstrated previously [33]. 

## Conclusion

In conclusion, our data support the notion that GK rats exhibit functional, metabolic and genomic differences in FA utilization independent of obesity. Functionally, GK rats exhibited structural remodeling and impaired cardiac function. Metabolically, GK rats exhibited increased FA utilization measured with quantitative metabolic imaging. The increase in FA utilization was primarily attributed to increased FA oxidation. Gene expression analysis on GK rats suggests a molecular signature of altered cardiac substrate preference, favoring increased fatty acid utilization. Taken together, the insights presented within may be used to devise targeted screening and therapeutic strategies for diabetes in the absence of the confounding factors of obesity, which are present in most animal models of Type 2 Diabetes.

## Supporting Information

Text S1 **In vivo quantification of myocardial fatty acid metabolism using [^11^C]Palmitate.**
(DOCX)

Figure S1 **Five-compartment, six-parameter model of [^11^C] Palmitate kinetics.** C_p_: Tracer concentration in plasma; C_1_: extracelluar tracer concentration; C_2_: cytosolic tracer concentration; C_3_: esterified tracer concentration; C_4_: oxidized tracer concentration; k_1_-k_5_, kinetic rate constants (min^-1^).
(TIF)

Figure S2 **Quantification of myocardial glucose utilization (MGU) in GK and Wistar rats.**
(TIF)

Figure S3 **Intrinsic measures of myocardial fatty acid metabolism as measured by PET.** (**A**) intrinsic myocardial fatty acid oxidation rate (MFAOUpR) (**B**) intrinsic myocardial fatty acid esterification rate (MFAEUpR), (**C**) intrinsic myocardial utilization (MFAUUpR) in GK and control rats. MFAOUpR, MFAEUpR and MFAUUpR represent the intrinsic capacity of the heart to oxidize, esterify and utilize fatty acids, respectively, independent of the concentration of free fatty acids in plasma. *denotes that GK rats are significantly different (P<0.05) than Wistars for that measurement. All results are presented as mean ± 1 SEM with N=4/group.
(TIF)
