# Supplementary material for: Genomic and Metabolic Disposition of Non-Obese Type 2 Diabetic Rats to Increased Myocardial Fatty Acid Metabolism
Source: PLoS One. 2013 Oct 21;8(10):e78477. doi: 10.1371/journal.pone.0078477 (PMC3804536; doi:10.1371/journal.pone.0078477)
Supplement: Text S1 — In vivo quantification of myocardial fatty acid metabolism using [11C]Palmitate. (DOCX) [file pone.0078477.s001.docx]

**Genomic and metabolic disposition of non-obese type 2 diabetic rats to increased myocardial fatty acid metabolism**

Sriram Devanathan^1^, Samuel T. Nemanich^1^, Attila Kovacs^2^, Nicole Fettig^1^, Robert J. Gropler^1, 3^, Kooresh I. Shoghi^1, 3, 4,*^

^1^Department of Radiology, ^2^Center for Cardiovascular Research, Department of Medicine, ^3^Department of Biomedical Engineering, and the ^4^Division of Biology and Biomedical Sciences, Washington University in St. Louis, Saint Louis, MO, 63110

**Supplemental Material**

*Estimation of Myocardial Blood Flow*

Myocardial blood flow (MBF; mL/g/min) was estimated using a 2 compartment, 2 parameter model describing the kinetics of [^11^C] Acetate in the heart. The rate constant k_1_ (mL/g/min), which is the turnover of tracer from blood to tissue, is optimized from the PET data, and is then scaled to calculate MBF as shown previously [[1](#_ENREF_1)].

*Estimation of Myocardial Glucose Metabolism*

At 18 weeks of age, FDG-PET imaging was performed to characterize myocardial glucose utilization (MGU) in GK and Wistar rats. The study design is described in the main manuscript. Regions of interest (ROI) were drawn similar to the description provided for analysis of [11C]Palmitate. Quantification of MGU was described previously [[2](#_ENREF_2)].

*Estimation of Myocardial Fatty Acid Metabolism*

Measures of myocardial fatty acid utilization (MFAU; nmol/g/min), esterification (MFAE, nmol/g/min) and oxidation (MFAO; nmol/g/min) were estimated using the five compartment, six parameter model (Figure S1), as we demonstrated previously [[1](#_ENREF_1),[3](#_ENREF_3)]. In brief, the concentration of tracer (mmol/g/L) in plasma is represented by C_p_(t). The remaining compartments, C_1_, C_2_, C_3_, and C_4_ represent the extracellular, cytosolic, esterified, and oxidized [^11^C]Palmitate respectively. The rate constants k_1_-k_5_ (min^-1^) denote the turnover between compartments. The parameter MBF/V (mL/g/min) is fixed, where V is the vascular fractional volume of the heart (10%) [[4](#_ENREF_4)]).

To calculate the oxidative rates, we consider the steady-state concentration of the system by setting the differential equations [1]-[4] to zero. The steady state concentration in C_2_(t), denoted by $C_{2}^{ss}$, is then used to calculate MFAU as a sum of MFAO and MFAE measures, where

$$\begin{matrix} MFAO=k_{5}C_{2}^{ss} \\ MFAE=k_{3}C_{2}^{ss} \end{matrix}$$

Thus, after estimating kinetic parameters k_1_-k_5_ from the PET data, we can calculate MFAO and MFAE from Eq. 5. The total utilization MFAU is then the sum of MFAO and MFAE. Additional measures reported in this work represent the intrinsic FA utilization (MFAU_UpR_; mL/g/min), oxidation (MFAO_UpR_; mL/g/min), and esterification (MFAE_UpR_; mL/g/min) rates in the heart, which are independent of circulating FA levels.

$$\begin{matrix} MFAU_{UpR}=\frac{MFAU}{[FFA]_{p}} \\ MFAO_{UpR}=\frac{MFAO}{[FFA]_{p}} \\ MFAE_{UpR}=\frac{MFAE}{[FFA]_{p}} \end{matrix}$$

where FFA_p_ denotes the concentration of FFA in plasma. Intrinsic measures of FA metabolism are denoted in Figure S3 whereas extrinsic measures are included in the manuscript. The extraction fraction (EF) characterizes the fraction of tracer extracted into heart tissue. It is calculated by

$$EF=\frac{MFAU_{UpR}}{MBF}$$

**Supplemental References**

1. Welch MJ, Lewis JS, Kim J, Sharp TL, Dence CS, et al. (2006) Assessment of myocardial metabolism in diabetic rats using small-animal PET: a feasibility study. J Nucl Med 47: 689-697.

2. Shoghi KI, Gropler RJ, Sharp T, Herrero P, Fettia N, et al. (2008) Time course of alterations in myocardial glucose utilization in the Zucker diabetic fatty rat with correlation to gene expression of glucose transporters: A small-animal PET investigation. Journal of Nuclear Medicine 49: 1320-1327.

3. Shoghi KI, Finck BN, Schechtman KB, Sharp T, Herrero P, et al. (2009) In vivo metabolic phenotyping of myocardial substrate metabolism in rodents: differential efficacy of metformin and rosiglitazone monotherapy. Circ Cardiovasc Imaging 2: 373-381.

4. Bergmann SR, Weinheimer CJ, Markham J, Herrero P (1996) Quantitation of myocardial fatty acid metabolism using PET. J Nucl Med 37: 1723-1730.
