# Supplementary figures and images for: Genomic and Metabolic Disposition of Non-Obese Type 2 Diabetic Rats to Increased Myocardial Fatty Acid Metabolism
Source: PLoS One. 2013 Oct 21;8(10):e78477. doi: 10.1371/journal.pone.0078477 (PMC3804536; doi:10.1371/journal.pone.0078477)

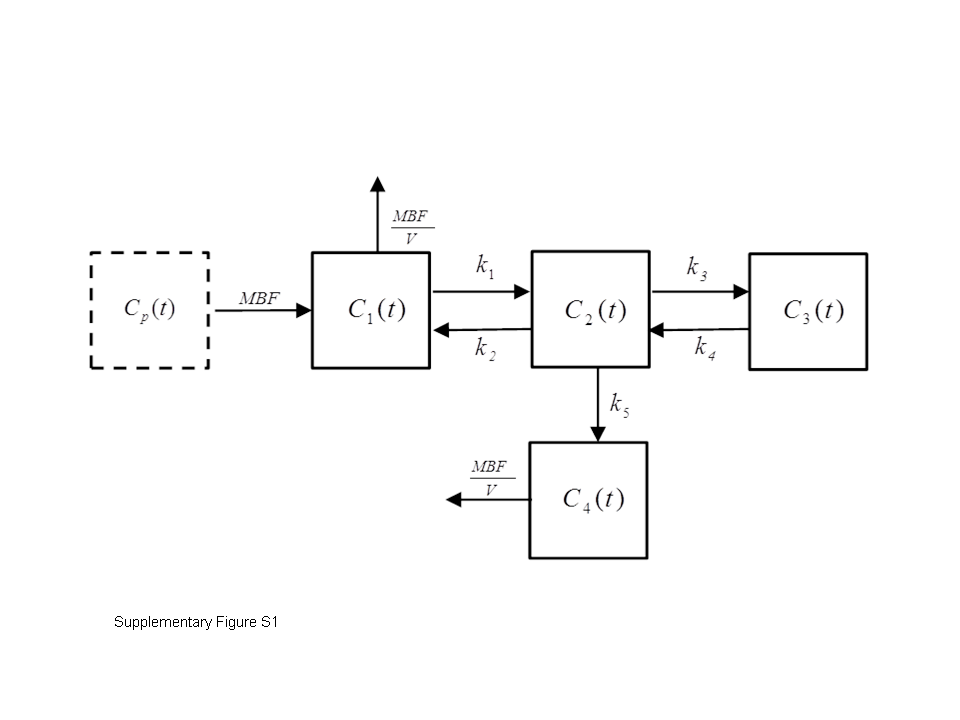

Supplement: Figure S1 — Five-compartment, six-parameter model of [11C] Palmitate kinetics. Cp: Tracer concentration in plasma; C1: extracelluar tracer concentration; C2: cytosolic tracer concentration; C3: esterified tracer concentration; C4: oxidized tracer concentration; k1-k5, kinetic rate constants (min-1). (TIF) [file pone.0078477.s002.tif]

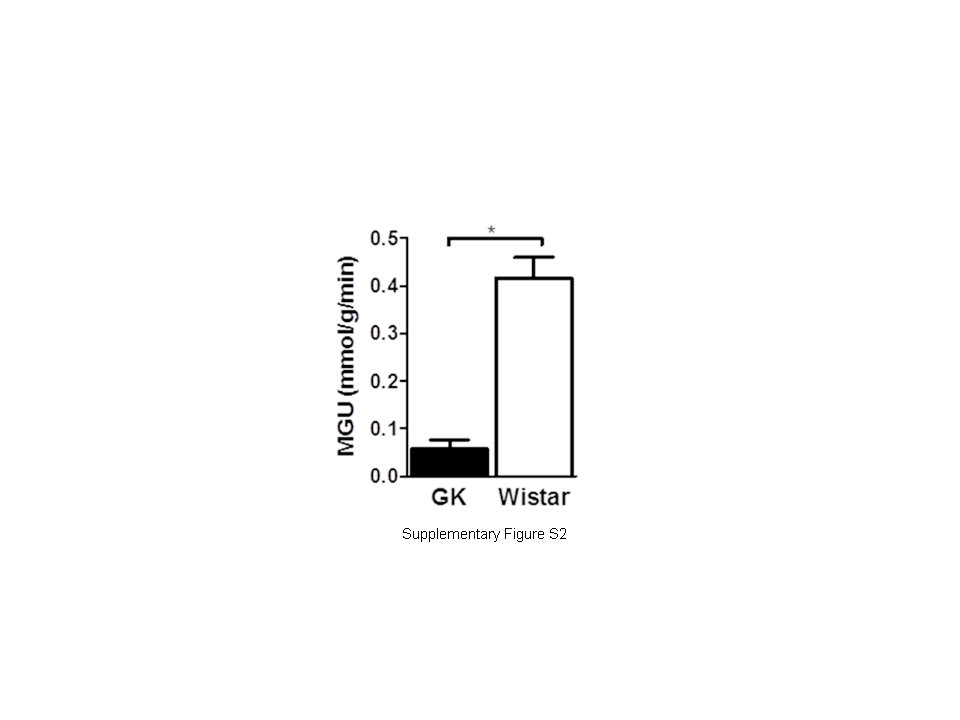

Supplement: Figure S2 — Quantification of myocardial glucose utilization (MGU) in GK and Wistar rats. (TIF) [file pone.0078477.s003.tif]

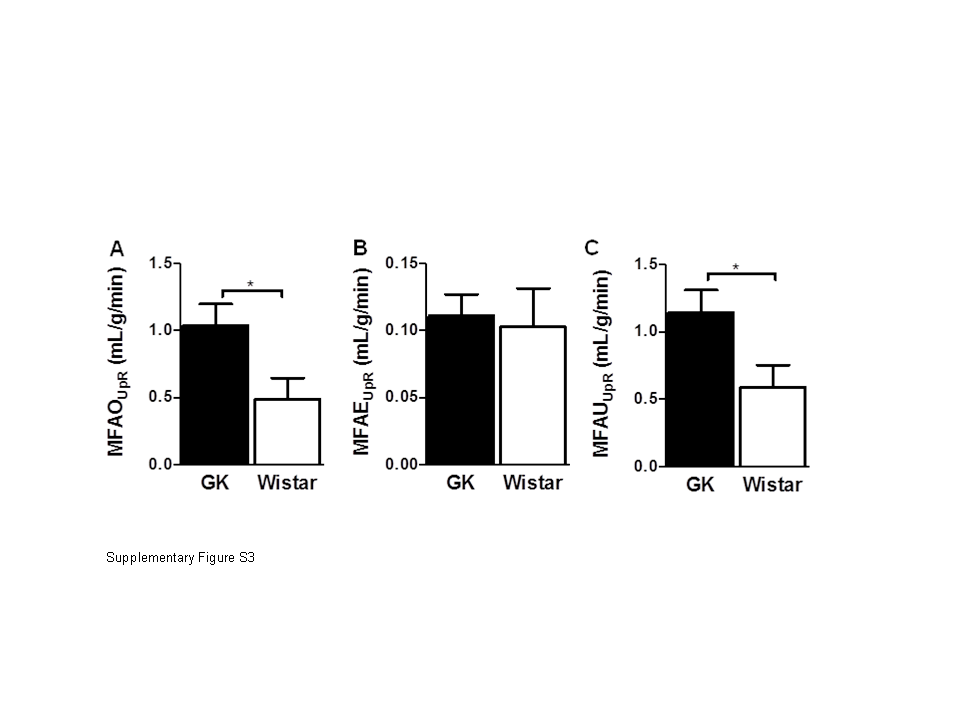

Supplement: Figure S3 — Intrinsic measures of myocardial fatty acid metabolism as measured by PET. (A) intrinsic myocardial fatty acid oxidation rate (MFAOUpR) (B) intrinsic myocardial fatty acid esterification rate (MFAEUpR), (C) intrinsic myocardial utilization (MFAUUpR) in GK and control rats. MFAOUpR, MFAEUpR and MFAUUpR represent the intrinsic capacity of the heart to oxidize, esterify and utilize fatty acids, respectively, independent of the concentration of free fatty acids in plasma. *denotes that GK rats are significantly different (P<0.05) than Wistars for that measurement. All results are presented as mean ± 1 SEM with N=4/group. (TIF) [file pone.0078477.s004.tif]
